# The role of glycerophospholipid metabolism in feline parvovirus infected CRFK cells

**DOI:** 10.3389/fmicb.2025.1658838

**Published:** 2025-08-22

**Authors:** Zhen Sun, Hongwei Zhu, Yang Liu, Jianlong Zhang, Linlin Jiang, Xin Yu, Jiayu Yu, Xingxiao Zhang

**Affiliations:** ^1^School of Life Sciences, Ludong University, Yantai, China; ^2^Collaborative Innovation Center for the Pet Infectious Diseases and Public Health in the Middle and Lower Stream Regions of the Yellow River, Yantai, China; ^3^Shandong Engineering Research Center for Aquaculture Environment Control, Yantai, China; ^4^Yantai Key Laboratory of Animal Pathogenic Microorganisms and Immunology, Yantai, China

**Keywords:** FPV013, PLPP1, Lpin3, glycerophospholipid metabolism, viral replication

## Abstract

**Background:**

Feline panleukopenia, caused by the highly lethal feline parvovirus (FPV), lacks effective prevention and treatment strategies. This study aimed to elucidate the key metabolic regulatory mechanisms during FPV infection.

**Methods:**

CRFK cells were infected with the FPV013 strain. Viral identification was performed via cytopathic effect (CPE) observation, transmission electron microscopy (TEM), PCR, and VP2 protein detection using Western blot and immunofluorescence. Untargeted metabolomics analyzed metabolic changes at 12 h, 24 h, and 48 h post-infection (hpi). Key pathways were validated through enzyme activity inhibition (Meclizine targeting PCYT2) and gene silencing (siRNA targeting PLPP1 and LPIN3).

**Results:**

Metabolomics revealed the most significant differences at 48 hpi, identifying six significantly altered glycerophospholipid metabolites. Inhibiting PCYT2 enzyme activity significantly reduced viral load (*p* < 0.01). Silencing either phospholipid phosphatase 1 (PLPP1) or LPIN3 significantly inhibited FPV replication, with IF staining showing reduced VP2 expression (*p* < 0.01). In contrast, blocking fatty acid synthesis (TOFA/C75 treatment) had no significant effect.

**Conclusion:**

FPV infection promotes replication by reprogramming host cell glycerophospholipid metabolism. The PCYT2-mediated PE/PC synthesis pathway and the PLPP1 or LPIN3 regulated diacylglycerol (DG) generation pathway play central roles. This finding highlights the pivotal role of glycerophospholipid metabolism during FPV infection, offering insights into antiviral strategies targeting this pathway.

## 1 Introduction

Feline parvovirus (FPV) is a single-stranded DNA virus, which belongs to the genus Protoparvovirus within the subfamily Parvovirinae of the family Parvoviridae (Allison et al., 2014). Feline panleukopenia, caused by feline parvovirus (FPV), is characterized by high incidence, mortality, and strong infectivity among felines (Allison et al., 2014; Kruse et al., 2010; Rehme et al., 2022). Infected cats exhibit symptoms including reduced appetite, vomiting, diarrhea, weight loss, fever, and weakness, with blood tests revealing significant leukopenia (Awad et al., 2018). Notably, FPV has been detected widely in canine populations across eastern and central China, with an infection rate of approximately 5% (Wang et al., 2022). Feline parvovirus (FPV) is typically transmitted via the fecal-oral route, with the main transmission pathways including contact with infected bodily fluids, feces, or other contaminated objects, as well as transmission through fleas (Stuetzer and Hartmann, 2014).

Metabolomics, the study of alterations in low-molecular-weight metabolites within cells or tissues, reflects responses to external stimuli and metabolic changes. This field has applications in disease diagnosis, drug development, nutrition and more. Genetic modifications are also pivotal for unraveling host cell interactions with external factors (Goldansaz et al., 2017). As obligate intracellular parasites, viruses hijack host cell metabolic machinery and resources for replication (Ghazal et al., 2000; Thaker et al., 2019). Analyzing metabolic flux in infected cells reveals shifts that can identify potential antiviral targets (Munger et al., 2008). For instance: HSV-1 stimulates aspartate synthesis to regulate pyrimidine nucleotide biosynthesis, involving increased glucose flux into the TCA cycle via pyruvate carboxylase (PC) and anaplerotic glutamine replenishment. Subsequent aspartate metabolism, facilitated by glutamic-oxaloacetic transaminase 2 (GOT2), promotes pyrimidine synthesis. Downregulating PC and GOT2 impedes HSV-1 replication. Furthermore, the lncRNA-ACOD1 enhances GOT2 activity; its absence reduces replication of HSV-1, vaccinia virus, and herpes simplex virus. Reducing aspartate-consuming enzymes like argininosuccinate synthase 1 (AS1) increases aspartate availability for viral replication, boosting viral titers (Grady et al., 2013; Oh et al., 2017). SARS-CoV-2 replication is linked to intracellular phosphatidic acid phosphatase I, and inhibiting this pathway curtails viral replication (Yan et al., 2022).

Metabolic alterations induced by viruses vary significantly. In this study, we employed a non-targeted metabolomic approach to identify metabolic shifts during FPV infection. A key finding was increased levels of phosphatidylcholine (PC) and phosphatidylethanolamine (PE), two predominant glycerophospholipids in mammalian cells, suggesting their significance in the FPV life cycle. PE synthesis occurs primarily via the CDP-ethanolamine Kennedy pathway and the phosphatidylserine (PS) decarboxylation pathway in mitochondria. PC synthesis involves multiple pathways: The CDP-choline pathway: Choline is phosphorylated by cytoplasmic choline kinase (CK), followed by conversion to CDP-choline by CTP: phosphocholine cytidylyltransferase (CT). CDP-choline then combines with diacylglycerol (DG) to form PC. PE methylation: PE can be converted to PC via three consecutive methylation reactions (Arii et al., 2020; Gohil et al., 2013; Pavlovic and Bakovic, 2013; Schenkel et al., 2015; Vance and Tasseva, 2013). Crucially, our study demonstrated that inhibiting PCYT2 enzyme activity significantly reduced the replication ability of FPV013, silencing PLPP1 or LPIN3 genes also inhibited viral proliferation, while blocking fatty acid synthesis had no obvious effect on viral replication. In conclusion, FPV013 infection can cause metabolic reprogramming in CRFK cells, in which the glycerophospholipid metabolic pathway (such as PCYT2-mediated PE/PC synthesis and PLPP1 or LPIN3-involved DG generation) is crucial for viral replication, whereas the fatty acid synthesis pathway shows low dependency. This observation underscores the pivotal role of glycerophospholipid metabolism in the context of FPV infection, providing valuable insights for potential antiviral strategies targeting this pathway.

## 2 Materials and methods

### 2.1 Cells and virus

CRFK cells, procured from Wuhan Procell Life Technology Co., Ltd. were cultured in Dulbecco’s Modified Eagle Medium (DMEM) supplemented with 10% fetal bovine serum (FBS) (ZETA, San Francisco, USA) and 1% penicillin/streptomycin at 37 °C and 5% CO_2_. The feline parvovirus (FPV013) was isolated from the anal swab of a cat with feline panleukopenia in the laboratory. Feline Parvovirus (FPV013) was maintained as the median tissue culture infection dose (TCID50) on CRFK cells, with a titer of 1.5 × 10^6^ TCID_50_/mL. The FPV positive control virus is isolated and identified from our laboratory, namely FPV072 (Xie et al., 2024).

### 2.2 Virus infection

CRFK cells into 6-well plates (1.6 × 10^6^ cells/well, 2 mL), and simultaneously inoculate FPV013 at a multiplicity of infection (MOI) of 0.0025. Incubate statically at 37 °C with 5% CO_2_ for 96 h.

### 2.3 Virus growth dynamics

After digesting the cells, the cell concentration was adjusted to 4 × 10^5^ cells/mL. 100 μL of the cell suspension was added to each well of a 96-well plate, which was then placed in a cell incubator at 37 °C with 5% CO_2_ for static culture. After 16 h, the cells grew into a single layer and were in good condition. The original medium was discarded, and the cells were washed twice with sterile PBS for later use. The harvested virus was serially diluted 10-fold up to 10^−8^ with diluent (DMEM + 1% penicillin–streptomycin) in microcentrifuge tubes and kept on ice. The diluted virus solutions were inoculated into the 96-well plate with 8 replicate wells for each dilution, 100 μL per well. The same volume of diluent was added to the negative control wells. After 1 h of incubation for infection, the viral supernatant was removed, and 200 μL of maintenance medium (DMEM + 2% FBS + 1% penicillin–streptomycin) was added to each well, followed by further static culture in the incubator. The cytopathic effects were observed, and the number of affected wells was recorded. The 50% tissue culture infectious dose (TCID_50_) was calculated using the Reed-Muench formula, and a growth curve was plotted.

### 2.4 Extracting the viral genome

The Feline Parvovirus (FPV) dye-based quantitative fluorescence PCR kit (Brand: Beijing Tianenze Gene Technology Co., Ltd., CAT#: 14-21400, V1.0) includes Fluorescent PCR-specific Template Diluent (Code: 180701), PCR Primer Mixture (14-21400yw), Positive Control (14-21400pc, 1 × 10⁸ copies/μL), and Nucleic Acid Release Reagent (61202). Specific procedures shall be performed according to the instructions in the kit manual.

### 2.5 Untargeted metabolomics analysis

CRFK cells co-infected with FPV013 (MOI = 0.0025) underwent LC–MS analysis at 0 h, 12 h, 24 h, and 48 h post-infection by Majorbio (Shanghai, China). The samples were analyzed by LC–MS/MS using the UHPLC-Q Exactive HF-X system (Thermo Fisher Scientific) at Shanghai Majorbio Biopharm Technology Co., Ltd. After LC–MS analysis, the raw data were imported into Progenesis QI (Waters Corporation, Milford, USA) for baseline filtering, peak identification, integration, retention time correction, and peak alignment to generate a data matrix containing retention time, mass-to-charge ratio, and peak intensity. Meanwhile, MS and MS/MS spectral data were matched against public databases (HMDB^1^; Metlin^2^) and the in-house database of Majorbio to annotate metabolites. The processed data matrix was uploaded to the Majorbio Cloud Platform^3^ for further analysis. First, data preprocessing steps were performed: (1) Application of the 80% rule to remove missing values (retaining variables with non-zero values in at least 80% of samples in each group); (2) Imputation of missing values using the minimum value from the original matrix; (3) Normalization of peak intensities via sum normalization to reduce errors from sample preparation and instrument variability; (4) Removal of variables with relative standard deviation (RSD) > 30% in QC samples; (5) Log10 transformation of the data. Next, principal component analysis (PCA) and orthogonal partial least squares discriminant analysis (OPLS-DA) were performed using the ropls package (Version 1.6.2) in R. Model stability was evaluated via 7-fold cross-validation. Metabolites with variable importance in projection (VIP) > 1 and *p* < 0.05 (Student’s *t*-test) were considered significantly different. These metabolites were annotated using the KEGG database^4^ for pathway mapping. Pathway enrichment analysis was conducted using the scipy.stats package in Python, and Fisher’s exact test was applied to identify significantly enriched biological pathways associated with experimental treatments. Metabolomics raw data have been deposited in MetaboLights: MTBLS12667.

### 2.6 Cell viability was measured by adding different drugs

Cells were digested and centrifuged according to the cell passaging method, resuspended in complete medium (DMEM + 10% FBS + 1% penicillin–streptomycin), and counted using a cell counter. CRFK cells were seeded into 96-well plates at 1 × 10^4^cells per well, followed by addition of different concentrations of Meclizine (25 μM, 50 μM, 100 μM), TOFA (5 μg/mL, 10 μg/mL, 20 μg/mL), C75 (2 μg/mL, 4 μg/mL, 8 μg/mL), or DMSO. After incubation at 37 °C with 5% CO_2_ for 24 h and 48 h, 10 μL of CCK-8 (CCK-8, Solarbio, Beijing, China) solution was added to each well, and incubation continued for 3 h. Absorbance was measured at 450 nm to calculate cell viability (%).

### 2.7 Meclizine treatment

To validate the inhibitory effect of Meclizine on the PCYT2 gene in CRFK cells, non-toxic drug concentrations were used for cell treatment. Cells were digested and centrifuged following standard passaging protocols, resuspended in maintenance medium (DMEM + 2% FBS + 1% penicillin–streptomycin), and counted using a cell counter. CRFK cells were seeded into 6-well plates at 1.6 × 10^6^ cells per well, co-infected with FPV013 (MOI = 0.0025), and treated with 50 μM Meclizine. An equal volume of DMSO was added to the control group. Infections were carried out at 37 °C with 5% CO_2_ for 24 h.

### 2.8 Inhibition of fatty acid synthesis in CRFK cells

To inhibit fatty acid synthesis in CRFK cells: Digest and centrifuge cells following the cell passage protocol, resuspend cells in maintenance medium (DMEM + 2% FBS + 1% penicillin–streptomycin), and perform cell counting using a cell counter. Seed CRFK cells into 6-well plates at a density of 1.6 × 10^6^ cells per well, and add TOFA at a final concentration of 5 μg/mL, C75 at a final concentration of 5 μg/mL, or an equal volume of DMSO. Inoculate FPV013 at an MOI of 0.0025, and incubate for 48 h at 37 °C in a 5% CO_2_ atmosphere.

### 2.9 Inhibition of diacylglycerol synthesis in CRFK cells

To inhibit diacylglycerol synthesis in CRFK cells and thereby affect the synthesis of phosphatidylethanolamine and phosphatidylcholine, we first verified the silencing of the phospholipid phosphatase 1 (PLPP1) gene through the following steps: For siRNA transfection, cells were digested and centrifuged following standard cell passaging procedures, resuspended in maintenance medium (DMEM + 2% FBS + 1% penicillin–streptomycin), and counted using a cell counter. CRFK cells were then seeded into 6-well plates at a density of 8 × 10^5^ cells per well and incubated at 37 °C with 5% CO^2^ for 16 h. Lipofectamine 3000 reagent (Invitrogen) was diluted in Opti-MEM medium (4.5 μL Lip3000 per 125 μL Opti-MEM), mixed 1:1 with 2.5 μg siRNA diluted in 125 μL Opti-MEM, and incubated at room temperature for 10 min. The resulting 250 μL DNA-lipid complex was then evenly added dropwise to the cells. In this experiment, PLPP1 and LPIN3 were silenced using siRNA sequences listed in Table 1, which were synthesized by Gemma Gene Biotechnology Company.

**Table 1:** siRNA sequences.

Gene	Sequence(5′-3′)
siNC-sense	UUCUCCGAACGUGUCACGUTT
siNC-antisense	ACGUGACACGUUCGGAGAATT
siPLPP1-sense	GGGUCUUUCUCGAGUUUCUTT
siPLPP1-antisense	AGAAACUCGAGAAAGACCCTT
siLPIN3-sense	GCAAGAAGGUGCCAAUGAUTT
siLPIN3-antisense	AUCAUUGGCACCUUCUUGCTT

### 2.10 Total RNA extraction

Total RNA was extracted using the phenol-chloroform method with minor modifications. Briefly, CRFK cells were lysed directly in culture plates by adding 1 mL of TRIzol reagent (Sigma-Aldrich, Germany) per well. The lysate was transferred to RNase-free microcentrifuge tubes and incubated at room temperature for 5 min to ensure complete dissociation of nucleoprotein complexes. 200 μL of chloroform was then added, the tubes were vortexed vigorously for 15 s, and incubated at room temperature for 3 min. Following centrifugation at 12,000×*g* for 15 min at 4 °C, the upper aqueous phase containing RNA was carefully transferred to a new tube. 500 μL of isopropyl alcohol was added to precipitate RNA, and the mixture was incubated at room temperature for 10 min before centrifugation at 12,000×*g* for 10 min at 4 °C. The RNA pellet was washed twice with 1 mL of 75% ethanol (prepared with DEPC-treated water), centrifuged at 7,500×*g* for 5 min at 4 °C, and air-dried for 5–10 min. The RNA was dissolved in 20 μL of DEPC-treated water and stored at −80 °C. RNA purity (A_260_/A_280_ ratio) and concentration were measured using a NanoDrop ND-1000 UV Vis spectrophotometer (Thermo Fisher Scientific, USA). Only samples with an A_260_/A_280_ ratio between 1.8 and 2.0 were used for subsequent experiments. cDNA was synthesized from 1 μg of total RNA using the Revert Aid First Strand cDNA Synthesis Kit (Thermo Scientific, USA) according to the manufacturer’s protocol. The reaction mixture included 5 × Reaction Buffer, 10 mM dNTP Mix, Random Hexamer Primers, and Revert Aid Reverse Transcriptase, with a final volume of 20 μL. The reaction was incubated at 25 °C for 10 min, followed by 42 °C for 60 min, and terminated at 70 °C for 5 min. The cDNA was diluted 10-fold with nuclease-free water and stored at −20 °C.

### 2.11 Quantitative real-time PCR (qPCR)

Quantitative PCR was performed using the SYBR Green Master Mix (Vazyme, Nanjing, China) on a QuantStudio 6 Flex Real-Time PCR System (Thermo Fisher Scientific, USA). The reaction system (total volume 20 μL) contained 10 μL of 2 × SYBR Green Master Mix with 0.4 μM of each primer; the list of primers is shown in Table 2. 2 μL of diluted cDNA template, and nuclease-free water. The thermal cycling conditions were as follows: 95 °C for 5 min (initial denaturation), followed by 40 cycles of 95 °C for 10 s (denaturation) and 60 °C for 30 s (annealing/extension). A melting curve analysis (60–95 °C, with a heating rate of 0.5 °C per second) was performed to confirm specific amplicon generation and exclude primer dimer formation. Relative gene expression levels were calculated using the 2^−ΔΔCt^ method, with normalization to the housekeeping gene GAPDH. Each sample was analyzed in technical triplicates, and results were presented as the mean ± standard deviation (SD).

**Table 2:** Primer sequences.

Gene	Sequence (5′-3′)
PLPP1-F	GCCTATGGCTGTTCTAAATTTGGGC
PLPP1-R	TGGGTAAACCAAGCCCCACT
LPIN3-F	CAGACAGTGAGCCTGAAGCCAT
LPIN3-R	AGCTCCTCAGAACTGGAATTGG
GAPDH-F	GCCGTGGAATTTGCCGT
GAPDH-R	GCCATCAATGACCCCTTCAT

### 2.12 Phosphoethanolamine transferase 2 (PCYT2) ELISA

The Phosphoethanolamine Transferase 2 (PCYT2) ELISA Kit (ST-H11004, Sentai, Shanghai Sentai Biotechnology Co., Ltd.) utilizes purified antibodies coated onto microplate wells to create a solid-phase antibody. Samples, standards, and horseradish peroxidase (HRP)-labeled detection antibodies are sequentially added to the coated wells. Following a wash step, the substrate 3,3′,5,5′-Tetramethylbenzidine (TMB) is added for color development. TMB is catalyzed by peroxidase to produce a blue color, which turns yellow upon acidification. The color intensity is directly proportional to the concentration of the target analyte in the sample. The absorbance (Optical Density, OD) is measured at 450 nm using a microplate reader, and the sample concentration is calculated based on the standard curve. Specific procedures should be performed according to the manufacturer’s instructions provided with the kit.

### 2.13 Statistical analysis

Statistical comparisons between the two groups were performed using an unpaired Student’s *t*-test. *p* < 0.05 was considered statistically significant. All statistical analyses were conducted using GraphPad Prism 8 software (GraphPad Software, San Diego, CA, USA).

## 3 Results

### 3.1 Identification of feline parvovirus

**Figure 1:**
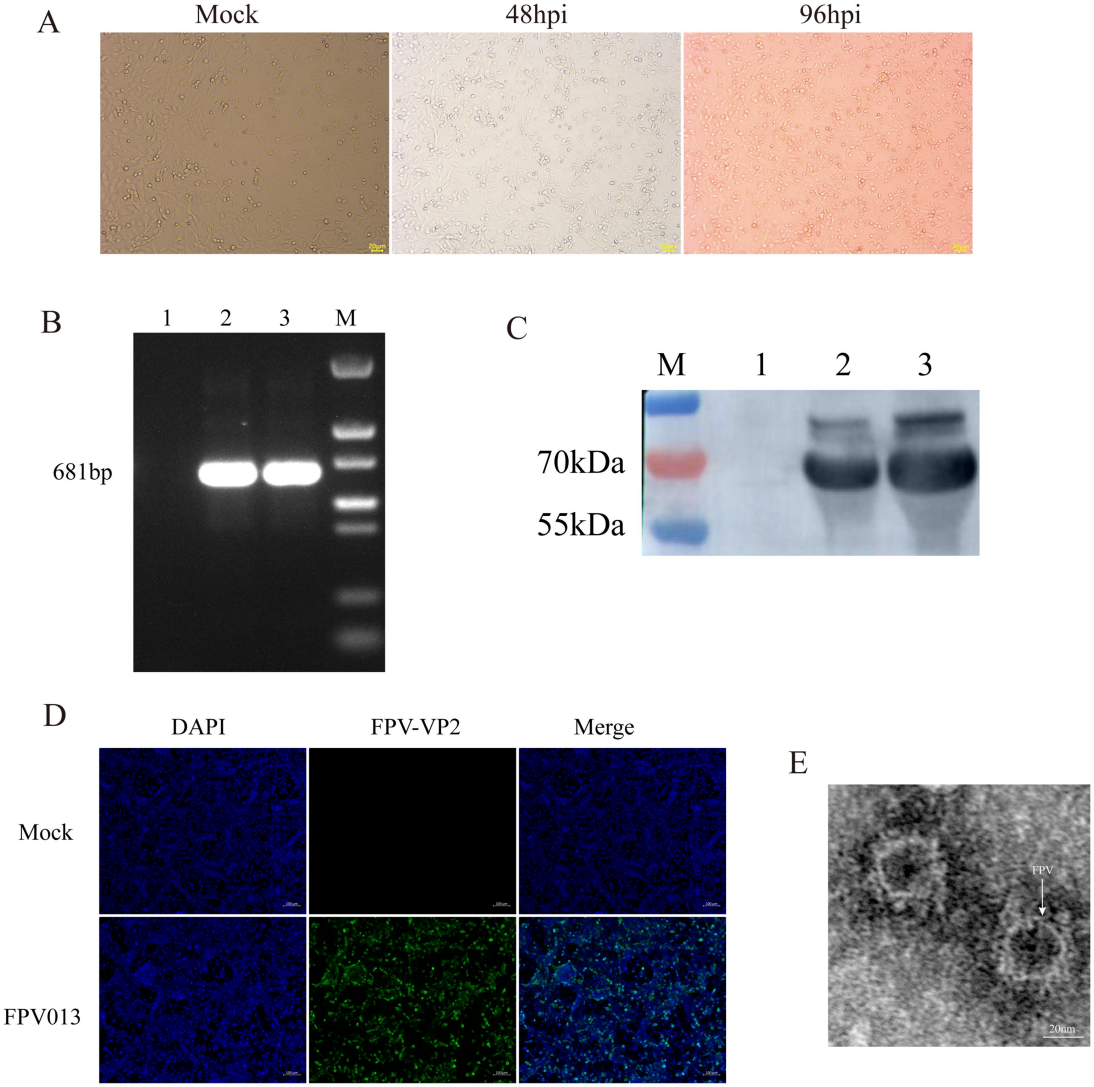
Identification of feline parvovirus. **(A)** Inoculation of CRFK cells with FPV013 resulted in the manifestation of cytopathic effects. MOCK: The simulated infection group was not inoculated with FPV; 48 hpi: Cells infected with FPV for 48 h; 96 hpi: Cells infected with FPV 96 h. **(B)** The FPV013 strain was identified by PCR. 1: Negative control, 2: Harvested virus supernatant, 3: Positive control, M: Marker. **(C)** The FPV013 virus species was identified by Western Blot. 1: Negative control, 2: Harvested virus supernatant, 3: Positive control, M: Page ruler. **(D)** Immunofluorescence identification of cells infected with FPV013. **(E)** Electron microscope results of FPV013.

**Figure 2:**
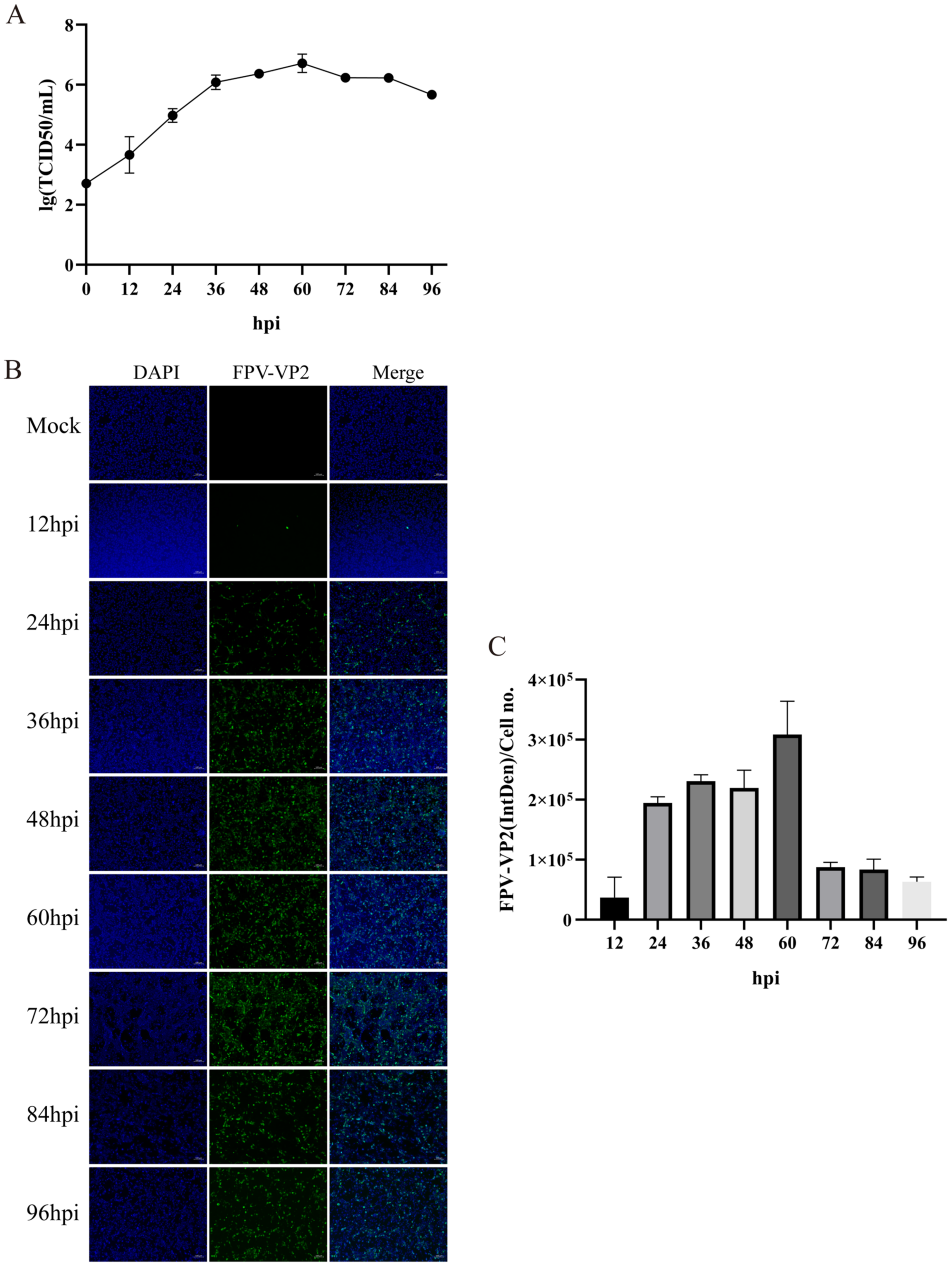
Growth kinetics of feline parvovirus. **(A)** One-step growth curves were plotted by TCID50 at different times. **(B)** Detection of FPV VP2 expression by indirect immunofluorescence assay (IFA). The scale bar represents 100 μm. **(C)** Calculation of the fluorescence ratio. Error bars indicate the standard deviation (SD) based on three independent experiments (n = 3). Statistical significance was determined using Student’s t-test (***p < 0.001, ****p < 0.0001).

Following infection of CRFK cells with FPV013 strain, significant cytopathic effects (CPE) were observed (Figure 1A). Compared to the mock-infected control group, partial cell rounding, shrinkage, and elongation were evident at 48 h post-infection (hpi) (Figure 1A). Prolonged infection to 96 hpi resulted in filamentous cellular morphology (Figure 1A). Primers targeting the VP2 gene of feline parvovirus (FPV) were designed for PCR amplification. Agarose gel electrophoresis analysis revealed a distinct band at approximately 681 bp in Lane 2, corresponding to the FPV013 sample (Figure 1B). This band aligned precisely with the positive control in Lane 3, confirming successful amplification of the VP2 gene fragment (Figure 1B). Immunoblotting of FPV013-infected cell lysates detected a prominent protein band at approximately 65 kilodaltons (kDa) in Lane 2, consistent with the positive control in Lane 3 (Figure 1C). This band corresponds to the expected molecular weight of the VP2 capsid protein, further validating the identity of FPV013. At 48 hpi, immunofluorescence microscopy of FPV013-infected CRFK cells exhibited intense fluorescence signals, in stark contrast to the mock-infected group, indicating robust viral protein expression (Figure 1D). TEM imaging of FPV013 virions revealed spherical, uniform particles approximately 25 nm in diameter, characteristic of parvovirus morphology (Figure 1E). To observe the replication of FPV013 in CRFK cells, the FPV013 strain was used to plot a one-step growth curve by TCID_50_ at different time points. The results showed that within 0–60 h post infection, the virus replication increased with the extension of time; the replication decreased within 60–72 h; the virus replication tended to be stable within 72–84 h; and the virus replication started to decline at 84 h (Figure 2A). To observe the replication of FPV013 in CRFK cells, after inoculating the CRFK cells with the FPV013 strain, the fluorescence intensity of single cells was detected at different time points (Figure 2B). The results showed that during the infection process, the fluorescence intensity of FPV VP2 reached a peak at 60 h (Figure 2C).

### 3.2 Non-target metabolic analysis of CRFK cells infected by FPV013

**Figure 3:**
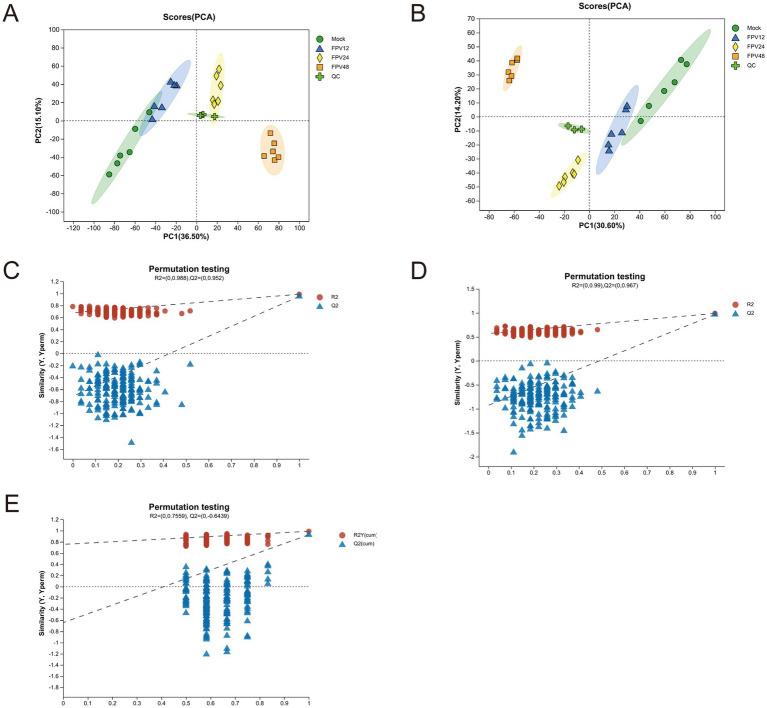
Multivariate analysis of metabolic profile. **(A)** PCA analysis of negative ionized metabolites; **(B)** PCA analysis of positive ionized metabolites; **(C)** PLS-DA analysis of negative ionized metabolites; **(D)** PLS-DA analysis of positive ionized metabolites; **(E)** OPLS-DA analysis of positive and negative ionized metabolites.

The original data were analyzed. Within 12 h, there was no obvious difference in peak intensity and peak shape between the base peak chromatograms of the blank group and the infected group. Differences began to appear at 24 h, and the most significant difference was observed at 48 h. Moreover, starting from 24 h after FPV013 infection, an obvious inter group dispersion trend was presented with the extension of time. It was speculated that the metabolic disorder was severe in the infected group at 48 h (Supplementary Figure S1). PCA, PLS-DA and OPLS-DA were used for the visualization of the analysis results. PCA analysis showed that the four groups were clearly distinguished, and the metabolic markers of CRFK cells infected by FPV013 presented an obvious inter-group dispersion trend (Figures 3A,B), indicating that the overall distribution trend of metabolites and the degree of difference between samples in each group of CRFK cells infected by FPV013 had changed. The QC samples were well aggregated, indicating that the system repeatability was stable. For the PLS-DA analysis of CRFK cells infected by FPV013 under positive and negative ion modes, in the negative ion mode, R2X = 0.7559 and Q2 = 0.6439; in the positive ion mode, R2X = 0.988 and Q2 = 0.952 (Figures 3C,D). There was an obvious distinction between each group, indicating that there were obvious differences in metabolites among each group of CRFK cells infected by FPV013. The further analyzed data were subjected to model verification, which showed that the Q2 intercept was less than 0, there was no over-fitting, and the model was reliable (Figure 3E).

**Figure 4:**
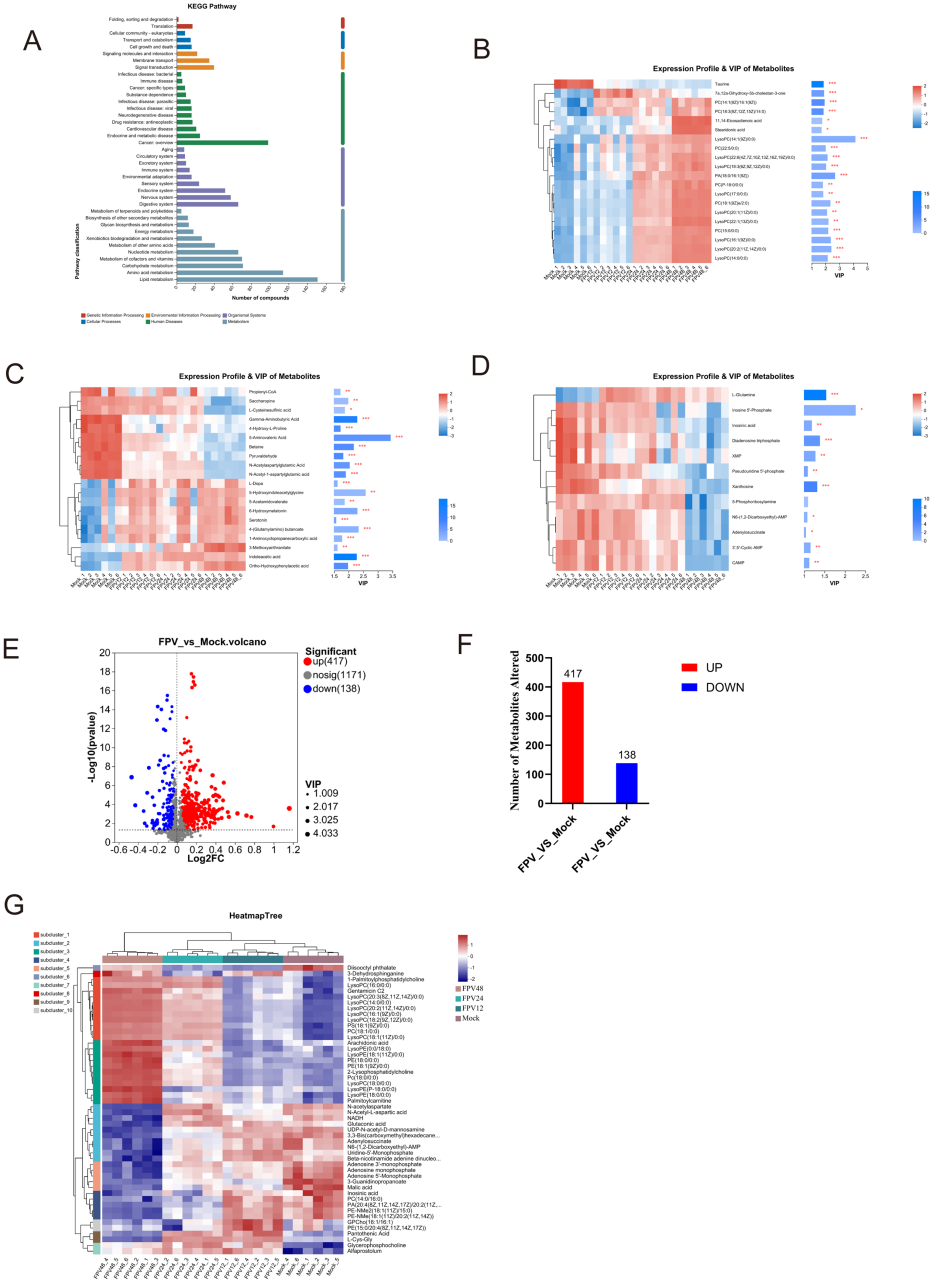
Cluster analysis of differential metabolites. **(A)** KEGG metabolic pathway; **(B)** Cluster heatmap of lipid metabolites; **(C)** Cluster heatmap of amino acid metabolites; **(D)** Cluster heatmap of nucleotide metabolites; **(E)** Volcano plot of FPV_vs_Mock. The x-axis represents Log_2_ fold change (Log_2_FC) of metabolite levels between FPV infected and mock infected samples. The y-axis shows—Log_10_ (*p* value) from statistical tests. **(F)** Bar graph quantifying the number of significantly altered metabolites in FPV_vs_Mock comparison. **(G)** HeatmapTree visualization of metabolite profiling across FPV infected and mock groups.

The infected and uninfected groups were compared using the VIP (Variable Importance in Projection) values. As shown in Supplementary Figure S2, both the x- and y-axis values were logarithmically transformed. Each point in the figure represents a specific metabolite, with its size reflecting the VIP value. Red points denote significantly upregulated metabolites, blue points indicate significantly downregulated metabolites, and gray points represent non-significant metabolites. The differential metabolites identified via MS^2^ matching were statistically analyzed (Supplementary Figures S2A,B). In the positive ion mode, after 12 h of FPV013 infection in CRFK cells, 195 metabolites were upregulated, and 71 were downregulated; after 24 h, 235 were upregulated, and 68 were downregulated; after 48 h, 197 were upregulated, and 120 were downregulated (Supplementary Figure S2C). In the negative ion mode, after 12 h, 164 metabolites were upregulated, and 107 were downregulated; after 24 h, 200 were upregulated, and 61 were downregulated; after 48 h, 183 were upregulated, and 124 were downregulated (Supplementary Figure S2C). KEGG pathway analysis revealed that lipid metabolism, amino acid metabolism, carbohydrate metabolism, cofactor and vitamin metabolism, and nucleotide metabolism contained the highest number of metabolites (Figure 4A). In this study, we performed cluster analysis on the top 20 metabolites with the highest abundance in lipid metabolism, amino acid metabolism, and nucleotide metabolism, respectively. Heatmaps were used to visualize the trends of differential metabolites across different groups. Following FPV013 infection in CRFK cells, the abundance of phosphatidylcholine (PC) species involved in biological membrane formation increased with prolonged infection time. 7α,12α-Dihydroxy-5β-cholestan-3-one, a cholesterol synthesis intermediate, was upregulated before 12 h post-infection (hpi) but downregulated thereafter. The abundance of unsaturated fatty acids such as 11,14-eicosadienoic acid and stearidonic acid increased progressively with infection duration (Figure 4B). Propionyl-CoA, associated with fatty acid metabolism, and pyruvaldehyde, a key product of amino acid metabolism, exhibited declining abundance over time. Conversely, metabolites related to tryptophan metabolism, including indoleacetic acid and 5-hydroxyindoleacetylglycine, showed increased abundance with prolonged infection (Figure 4C). Only glutamine abundance increased during FPV013 infection, while other nucleotide metabolites such as cyclic AMP (cAMP) and inosinic acid were downregulated (Figure 4D). Combining data from all-time points and both ion modes, we identified 417 upregulated and 138 downregulated metabolites, totaling 555 differential metabolites (Figures 4E,F). Cluster analysis of this metabolite set revealed that the MOCK group and infected groups formed distinct hierarchical clusters, with infection time points showing a progressive relationship. Left-side clustering grouped metabolites into 10 subclusters, with three subclusters (subcluster_8, subcluster_1, subcluster_3) showing close relationships and increasing metabolite abundance over time. These subclusters primarily contained phosphatidylcholine (PC) and phosphatidylethanolamine (PE), essential components of biological membranes (Figure 4G).

**Figure 5:**
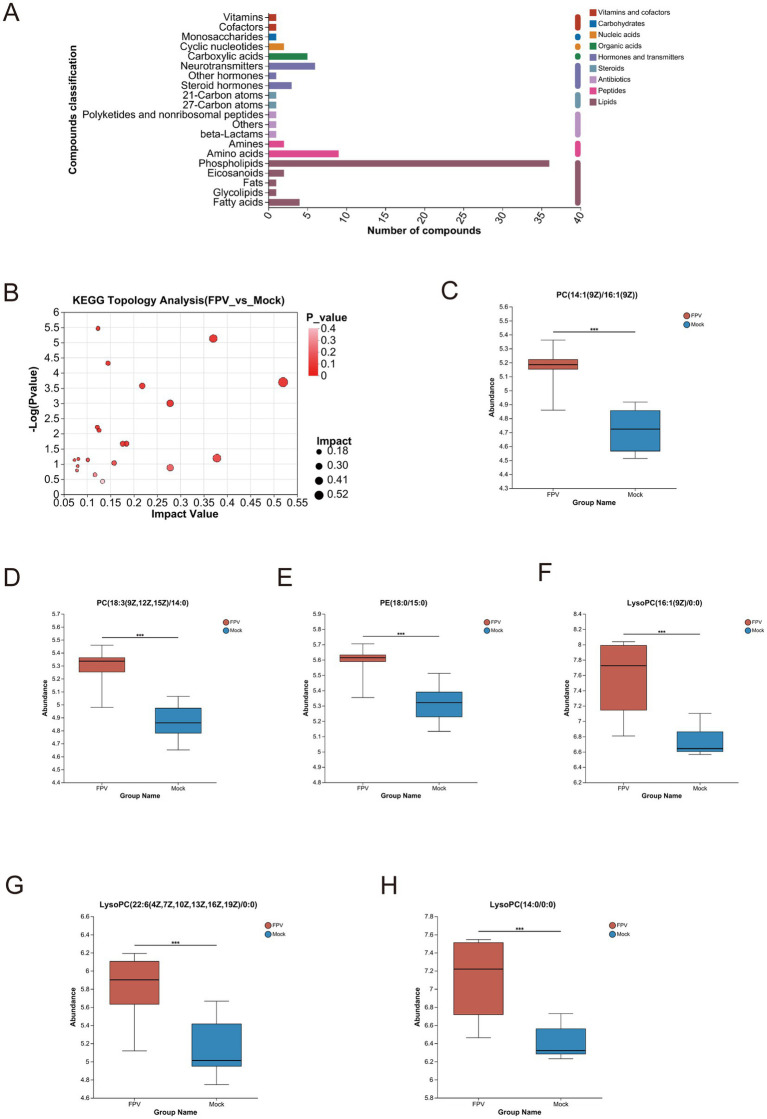
Differential metabolites and enriched pathways in cells infected with FPV013. **(A)** Enrichment analysis of differential metabolite pathways. **(B)** KEGG topological analysis. **(C–H)** Differential analysis of core metabolites. Error bars indicate the standard deviation (SD) based on three independent experiments (*n* = 6). Statistical significance was determined using Student’s t-test (***p < 0.001).

In this study, identified metabolites were classified according to their hierarchical biological functions. Metabolites were mapped to the KEGG Compound database to obtain a classification profile, which was statistically plotted. Cluster analysis was focused on lipid metabolism, compared with the MOCK group, differential metabolites in infected cells were mainly clustered in phospholipids (Figure 5A). A KEGG topological analysis was conducted on phospholipid-class differential metabolites (Figure 5B), and the results (as shown in the Table 3) indicated that the glycerophospholipid metabolism pathway was the most significantly enriched pathway. The identified phospholipids included two phosphatidylcholine (PC) conformations [PC (14:1(9Z)/16:1(9Z)) and PC (18:3(9Z,12Z,15Z)/14:0)], one phosphatidylethanolamine (PE) [PE (18:0/15:0)], and three lysophosphatidylcholine (LysoPC) conformations [LysoPC (16:1(9Z)/0:0), LysoPC (22:6(4Z,7Z,10Z,13Z,16Z,19Z)/0:0), and LysoPC (14:0/0:0)]. The abundance fluctuations of these six phospholipids were evaluated using box plots (Figure 5B). Among them, LysoPC (16:1(9Z)/0:0) and LysoPC (14:0/0:0) showed the highest abundance during infection but with greater fluctuations (Figures 5C–H).

**Table 3:** Enrichment of pathways.

Num	Match_status	Pathway description
36	5|52	Glycerophospholipid metabolism
1	1|21	Ether lipid metabolism
2	1|32	Glycerolipid metabolism
6	1|13	Linoleic acid metabolism
6	1|30	alpha-Linolenic acid metabolism
6	1|37	Arachidonic acid metabolism
1	1|47	Glycine, serine and threonine metabolism

### 3.3 The effect of glycerophospholipid metabolic pathway abnormality on FPV infection and replication

**Figure 6:**
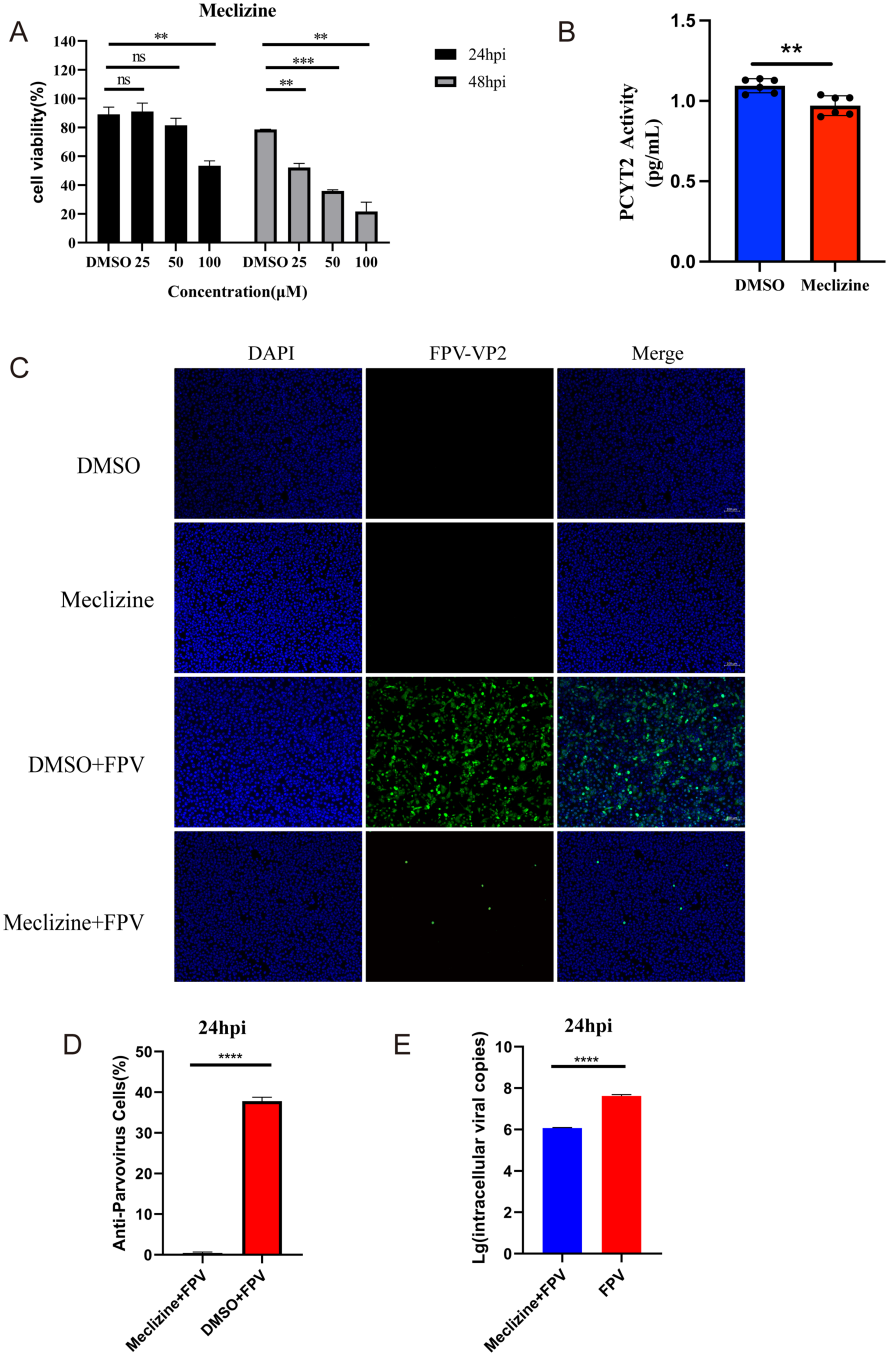
Effect of meclizine on FPV013 replication. **(A)** Detection of cytotoxicity of meclizine at different concentrations; **(B)** Real-time fluorescence levels were quantitatively detected to assess the expression of PCYT2 in CRFK cells treated with meclizine, DMSO, or infected with FPV. **(C)** Indirect immunofluorescence technique was used to detect the expression of FPV VP2, with a scale bar of 100 μm. **(D)** Calculation of the fluorescence ratio. **(E)** Copy number of FPV in CRFK cells after addition of Meclizine by qPCR. Error bars represent the standard deviation (SD) based on three independent experiments (*n* = 3). Statistical significance was determined using a t-test (**p < 0.01, ***p < 0.001).

Differential analysis of non-targeted metabolites, KEGG pathway enrichment, and topological analysis revealed a significant enhancement of lipid metabolism, particularly the glycerophospholipid metabolic pathway. During viral infection, aberrations in phospholipid metabolism may disrupt cellular functions and even facilitate viral replication and transmission (Yan et al., 2022). The PCYT2 gene, a key regulator in this pathway, encodes inositol phospholipid synthase, which is critical for phospholipid biosynthesis (Gohil et al., 2013). Conversely, the PAP-1/PLPP1 gene encodes phosphatidic acid phosphatase, a regulator of membrane phospholipid turnover and signal transduction (Yan et al., 2022). The study found that Meclizine can inhibit the enzymatic activity of PCYT2 through non-competitive inhibition. Does the inhibition of PCYT2 enzymatic activity affect the infection of CRFK cells by FPV013? First, the effect of adding different concentrations of Meclizine on the viability of CRFK cells was determined. After 24 h of drug treatment, compared with the DMSO control, the cell viability was higher than 80% when the drug concentration was lower than 50 μM, while the cell viability significantly decreased after 48 h of drug treatment (Figure 6A). To determine the inhibition of PCYT2 enzymatic activity in CRFK cells by Meclizine, CRFK cells were treated with a final concentration of 50 μM Meclizine for 24 h, and the PCYT2 enzymatic activity was significantly reduced (Figure 6B). During the infection of CRFK cells by FPV013, a final concentration of 50 μM Meclizine was added. After 24 h of infection, the expression of FPV VP2 was detected by immunofluorescence. The fluorescence rate in the Meclizine treatment group was significantly lower than that in the DMSO group (Figures 6C,D). The copy number of FPV013 in the Meclizine treatment group was significantly reduced as detected by the FPV probe method (Figure 6E). To determine whether inhibiting fatty acid biosynthesis affects the replication of FPV013 in CRFK cells, TOFA or C75 was added to CRFK cells, followed by inoculation with FPV013. TOFA acts on acetyl-CoA carboxylase to inhibit its activity, block the generation of malonyl-CoA, and thus suppress fatty acid synthesis. C75 acts on fatty acid synthase to hinder the elongation of fatty acid chains, reducing the production of fatty acids such as palmitic acid. First, the toxicity of different concentrations of TOFA/C75 to CRFK cells was tested. As shown in Supplementary Figures S3A,B, after 24 h of drug treatment, compared with the DMSO control, TOFA at concentrations not exceeding 5 μg/mL and C75 at concentrations not exceeding 2 μg/mL did not significantly affect cell viability. Then, final concentrations of 5 μg/mL TOFA and 2 μg/mL C75 were added, followed by inoculation with FPV013 to infect CRFK cells. After 24 h of infection, the expression of FPV013 VP2 was detected by immunofluorescence staining (Supplementary Figures S3C–E). The results showed that blocking fatty acid biosynthesis with inhibitors did not affect the replication of FPV013 in CRFK cells.

**Figure 7:**
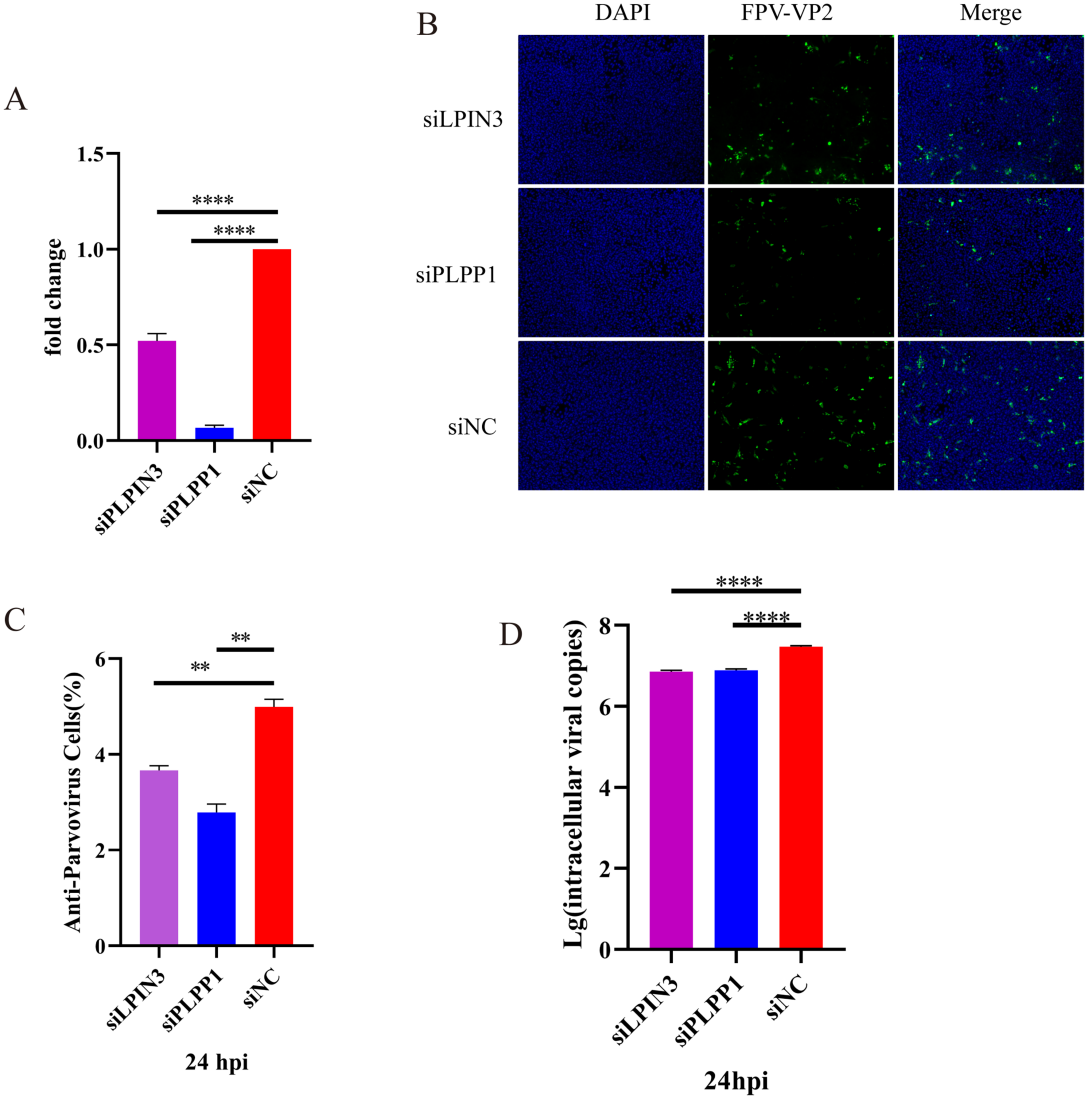
Effect of blocking the activity of phosphatidyl phosphatase on CRFK cells infected with FPV013. **(A)** Inhibitory effect of siRNA transfection on PLPP1 expression; **(B)** Indirect immunofluorescence technique was used to detect the expression of FPV VP2, with a scale bar of 100 μm; **(C)** Calculation of the fluorescence ratio. **(D)** Copy number of FPV in CRFK cells after deletion of LPIN3 or PLPP1 by qPCR. Error bars represent the standard deviation (SD) based on three independent experiments (*n* = 3). Statistical significance was determined using a t-test (**p < 0.01; ****p < 0.0001).

PLPP1/PAP-1 (phosphatidic acid phosphatase 1), a key enzyme in glycerophospholipid metabolism, catalyzes the hydrolysis of phosphatidic acid (PA) into diacylglycerol (DG) and inorganic phosphate, thereby directly regulating the glycerophospholipid synthesis pathway. The produced DG can be further converted into various glycerolipids such as triacylglycerol (TG), phosphatidylcholine (PC), and phosphatidylethanolamine (PE) through the glycerophospholipid metabolic pathway. PLPP1 (Phosphatidic Acid Phosphatase 1) represents the current standard gene/systematic nomenclature for this enzyme, as established by gene nomenclature committees (e.g., HGNC). This designation emphasizes its specific membership (member 1) within the phosphatidic acid phosphatase family (PLPP). PAP-1 (Phosphatidate Phosphatase 1) denotes the historical or functionally classified common name for this enzyme, predominantly used in earlier biochemical literature. Regardless of being referred to as PAP-1 or PLPP1, both terms identify the same key enzyme in glycerophospholipid metabolism, which catalyzes the hydrolysis of phosphatidic acid (PA) into diacylglycerol (DG) and inorganic phosphate (Pi). For consistency in terminology, the enzyme’s role will be uniformly represented by PLPP1 in the subsequent sections of the article. The lipin family comprises three lipin proteins (lipin 1, lipin 2, and lipin 3), all of which possess phosphatidic acid phosphatase activity. This enzymatic function enables them to catalyze the hydrolysis of phosphatidic acid (PA) into diacylglycerol (DG) and inorganic phosphate. Consequently, they serve as core enzymes regulating lipid synthesis and signaling pathways in mammalian cells (Zhang et al., 2019). To determine whether silencing of PLPP1 gene and blockade of glycerophospholipid metabolism pathway affect the replication of FPV013 in CRFK cells, siRNAs targeting PLPP1 and LPIN3 were used for gene silencing. First, RT-qPCR analysis showed that transfection of CRFK cells with siPLPP1 or siLPIN3 significantly reduced the expression of PLPP1 gene (Figure 7A). To assess the impact of impaired PLPP1 expression on FPV013 replication in CRFK cells, indirect immunofluorescence assay was performed to detect the expression of FPV VP2. The results showed that transfection of CRFK cells with siPLPP1 or siLPIN3 significantly decreased the fluorescence intensity of FPV VP2 (Figures 7B,C). Absolute quantification of FPV013 in infected CRFK cells was conducted by FPV probe-based real-time fluorescence quantitative PCR, demonstrating that silencing of PLPP1 gene led to a significant reduction in FPV load compared with the uninfected group (Figure 7D). These findings indicate that silencing of PLPP1 gene and subsequent blockade of glycerophospholipid metabolism pathway affect the replication of FPV013 in CRFK cells.

## 4 Discussion

In this study, CRFK cells were inoculated with FPV013, and obvious cytopathic effects (CPE) were observed, including cell rounding, contraction, and filamentation. FPV013 was preliminarily identified by PCR. However, due to the high homology between FPV and CPV, PCR-based identification alone has certain limitations. The VP2 protein of FPV contains amino acid types that determine host range. Therefore, in addition to molecular-level identification, VP2 protein expression was detected at the protein level. Western Blot and indirect Immunofluorescence (IF) techniques were used to confirm VP2 expression. Morphologically, the supernatant of FPV-infected CRFK cells was observed by transmission electron microscopy, and uniform spherical particles (~25 nm in diameter) were visualized in the field of view. To monitor FPV replication in CRFK cells, a one-step growth curve was established, and VP2 expression was analyzed at different time points. Results showed that viral titers were consistent with VP2 expression trends, though protein levels decreased at 48 h compared to 36 h but continued to increase from 48–60 h. Based on this fluctuating pattern, 12 h, 24 h, and 48 h were selected for metabolite analysis. Additionally, the IF technique provided a more intuitive reflection of VP2 expression, supporting method validation for subsequent analyses.

This study explored the effects of FPV013 infection on the metabolism of CRFK cells using liquid chromatography-mass spectrometry (LC–MS) profiling and multivariate analysis. Significant metabolic differences were observed between infected and uninfected groups, with the most pronounced variations detected at 48 h post-infection, indicating that FPV013 infection induces comprehensive metabolic changes in CRFK cells that intensify over time. Multivariate analysis revealed distinct inter-group dispersion of metabolic markers, suggesting significant alterations in the overall distribution and diversity of metabolites. These findings imply that FPV013 infection triggers metabolic network reprogramming in CRFK cells, potentially as a physiological adaptive response to viral infection. Screening of differentially expressed metabolites identified substantial changes, particularly an increase in phospholipid abundance with prolonged infection. These changes reflect the impact of viral infection on key biological processes such as cell membrane composition and signal transduction. Differential analysis of metabolites in FPV-infected CRFK cells primarily identified phospholipid molecules, with six glycerophospholipids (GPLs) screened: PC(14:1(9Z)/16:1(9Z)), PC(18:3(9Z,12Z,15Z)/14:0), PE(18:0/15:0), LysoPC(16:1(9Z)/0:0), LysoPC(22:6(4Z,7Z,10Z,13Z,16Z,19Z)/0:0), and LysoPC(14:0/0:0), highlighting the critical role of the glycerophospholipid metabolic pathway during FPV infection. Glycerophospholipids (GPLs), including phosphatidylcholine (PC) and phosphatidylethanolamine (PE), are among the most abundant lipid types in cell membranes. These phospholipids are essential for maintaining membrane structure and function in mammalian cells. Elucidating the glycerophospholipid metabolic pathway provides insights into membrane construction and regulatory mechanisms, offering theoretical foundations for research and therapy of related diseases (Hermansson et al., 2011).

Phosphatidylcholine (PC) serves as a major component of mammalian cell membranes, whereas phosphatidylethanolamine (PE) predominates in purified viral particles [84]. As a key rate-limiting enzyme in the PE synthesis pathway, PCYT2 (phosphatidylcholine cytidylyltransferase 2) directly maintains PE biosynthesis by regulating the production of CDP-ethanolamine (CDP-Etn) and indirectly influences PC replenishment, acting as a critical hub connecting ethanolamine metabolism with membrane phospholipid (PE/PC) biosynthesis (Hermansson et al., 2011; Lykidis et al., 2001; Pavlovic and Bakovic, 2013). In this study, meclizine was used to inhibit PCYT2 enzymatic activity and disrupt normal PE synthesis. Experiments showed that adding meclizine to CRFK cells significantly suppressed PCYT2 activity. Following inoculation with FPV013 virus, indirect immunofluorescence staining for FPV VP2 protein and real-time fluorescent quantitative analysis with FPV-specific probes confirmed that both the replication capacity and viral load of FPV013 in CRFK cells were significantly reduced. These results indicate that the PCYT2-mediated glycerophospholipid (PE/PC) metabolic pathway plays a decisive role in FPV013 replication. Inhibition of this pathway disrupts membrane phospholipid synthesis, thereby blocking viral proliferation.

In the glycerophospholipid metabolic pathway, diacylglycerol (DG), as a key lipid mediator, can be converted into various glycerolipids such as triacylglycerol (TG), phosphatidylcholine (PC), and phosphatidylethanolamine (PE) through this pathway. Notably, DG is generated by the specific catalysis of phosphatidic acid (PA) by PLPP1, which plays an indispensable role in mediating metabolic pathways and is closely associated with viral replication (Yan et al., 2022). In cisplatin-resistant lung cancer cells, downregulated expression of PLPP1 leads to the accumulation of PA and a reduction in DG (Geng et al., 2023). The expression and activity of PLPP1 are regulated by the LPINs gene family, and it participates in glycerophospholipid metabolism, glycerolipid metabolism, and the mTOR signaling pathway (Yan et al., 2022). Previous studies by Yan Bingpeng et al. have confirmed that silencing the PLPP1 gene significantly inhibits the replication of SARS-CoV-2, and the absence of lipin2 or lipin3 also exerts a marked inhibitory effect on SARS-CoV-2 replication (Yan et al., 2022). The lipin family consists of three members (lipin1, lipin2, and lipin3), all of which exhibit phosphatidic acid phosphatase (PAP) activity but show significant differences in tissue distribution. Specifically, lipin1 is highly expressed in metabolism-dominant tissues such as adipose tissue, heart, and skeletal muscle, whereas lipin2 and lipin3 are specifically enriched in non-metabolism-dominant tissues (e.g., intestine and brain) (Csaki et al., 2014). The feline kidney cells used in this study belong to atypical metabolic tissues, and their lipin expression profile more closely resembles that of lipin2/lipin3 dominant organs like the intestine. Therefore, we focused on investigating the function of lipin3. Based on these findings, we used siRNA technology to silence PLPP1 or LPIN3 genes in CRFK cells, followed by inoculation with FPV013 virus. Indirect immunofluorescence staining for FPV VP2 protein and real-time fluorescent quantitative analysis with FPV-specific probes confirmed that both the replication capacity and viral load of FPV013 in CRFK cells were significantly reduced. These results indicate that PLPP1 or LPIN3 genes likely play a positive regulatory role in FPV013 replication by participating in glycerophospholipid metabolism and related pathways, and their expression deficiency effectively inhibits viral proliferation in cells.

In addition to the glycerophospholipid metabolic pathway, fatty acid synthesis is also a critical process in cellular metabolism. In the experiment, TOFA (which inhibits acetyl-CoA carboxylase to block the initiation of fatty acid synthesis) and C75 (which inhibits fatty acid synthase to hinder chain elongation) were, respectively, added to CRFK cells, followed by inoculation with FPV013 and cultivation for 24 h. Immunofluorescence staining detection showed that blocking fatty acid biosynthesis did not affect the expression of FPV013 VP2 protein. This indicates that the proliferation of FPV013 in CRFK cells may not rely on *de novo* synthesized fatty acids in cells, and its replication process has a low dependency on the fatty acid synthesis pathway.

## 5 Conclusion

**Figure 8:**
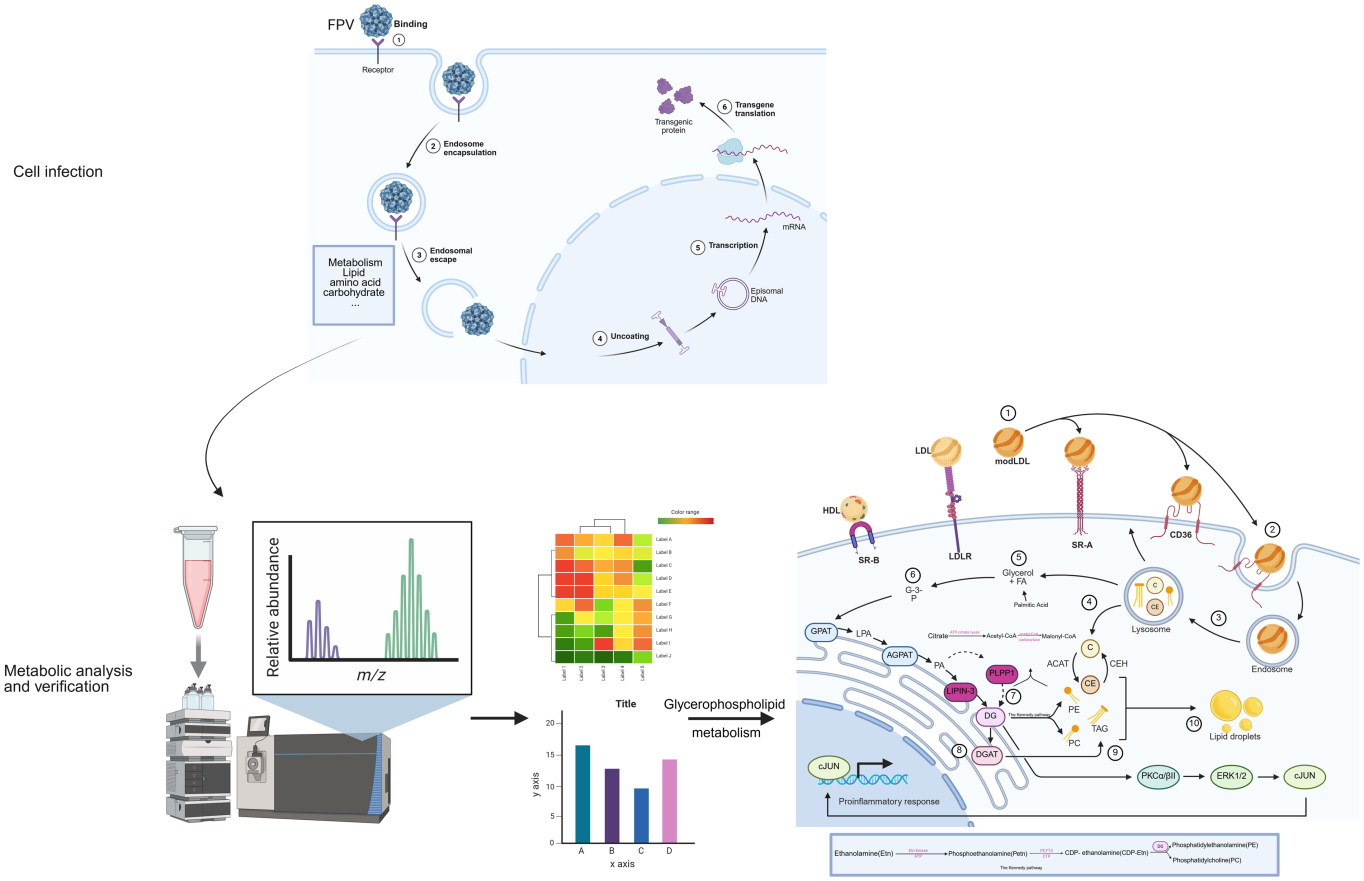
The process by which feline parvovirus (FPV) infects cells and induces metabolic regulation This schematic diagram systematically presents the process of feline parvovirus (FPV) infecting cells and inducing metabolic regulation. First, in the cell infection stage, FPV sequentially undergoes binding to cell receptors, endosomal encapsulation, endosomal escape, and uncoating. Subsequently, transcription and transgene translation occur, and at the same time, it triggers the reprogramming of lipid and carbohydrate metabolism in host cells. Next, through the metabolic analysis and verification process, samples of infected cells are collected and detected using liquid chromatography mass spectrometry technology. The relative abundance of metabolites is presented via mass to charge ratio (m/z) peak diagrams. Differential metabolite heatmaps are used for cluster analysis to screen core pathways, and then bar charts are employed to verify the abundance differences of key metabolites such as glycerophospholipids, anchoring the regulatory effect of infection on metabolic pathways. Finally, focusing on the glycerophospholipid metabolism regulatory network, it shows that lipoproteins enter cells via receptors to provide raw materials, enzymes such as AGPAT and LPIN mediate phospholipid synthesis and transformation, and metabolic intermediates activate the PKC-ERK1/2 pathway to regulate the release of pro-inflammatory factors, forming a “virus metabolism immunity” interaction. This reveals the mechanism by which FPV hijacks the host’s glycerophospholipid metabolism to support its own replication and affect cell functions [Created in BioRender. Hou, Y. (2025) https://BioRender.com/el3ej9c].

This study demonstrates that infection of CRFK cells with FPV013 induces cytopathic effects and reprograms host cell metabolism. Crucially, the glycerophospholipid metabolic pathway specifically involving PCYT2-mediated phosphatidylethanolamine/phosphatidylcholine (PE/PC) synthesis and PLPP1 or LPIN3-dependent diacylglycerol (DG) generation—plays an essential role in viral replication. In contrast, viral proliferation exhibits minimal dependence on the fatty acid synthesis pathway (Figure 8).

## Data Availability

Publicly available datasets were analyzed in this study. This data can be found here: Metabolomics raw data have been deposited in MetaboLights: MTBLS12667.

## Footnotes

^1^ http://www.hmdb.ca/

^2^ https://metlin.scripps.edu/

^3^ cloud.majorbio.com

^4^ https://www.kegg.jp/kegg/pathway.html
