# Supplementary material for: The role of glycerophospholipid metabolism in feline parvovirus infected CRFK cells
Source: Front Microbiol. 2025 Aug 22;16:1658838. doi: 10.3389/fmicb.2025.1658838 (PMC12411486; doi:10.3389/fmicb.2025.1658838)
Supplement: Supplementary file 1 [file Data_Sheet_1.docx]

**Supplementary Materials Methods**

**1 Virus Infection**
Digest and centrifuge cells according to the cell passage method, resuspend cells with maintenance medium (DMEM + 2% FBS + 1% penicillin-streptomycin), and perform cell counting using a cell counter. Inoculate CRFK cells into 6-well plates (1.6×10^6^ cells/well, 2 mL), and simultaneously inoculate FPV013 at a multiplicity of infection (MOI) of 0.0025. Incubate statically at 37°C with 5% CO_2_ for 96 hours. Harvest viruses at 12-hour intervals, freeze at -80°C for 2 hours, rapidly dissolve at 37°C, repeat three times, centrifuge at 3000 r/min for 30 minutes, collect viral supernatants, and store at -80°C.

**2 Extracting the viral genome**

The Feline Parvovirus (FPV) dye-based quantitative fluorescence PCR kit (Brand: Beijing Tianenze Gene Technology Co., Ltd., CAT#: 14-21400, V1.0) includes Fluorescent PCR-specific Template Diluent (Code: 180701), PCR Primer Mixture (14-21400yw), Positive Control (14-21400pc, 1×10⁸ copies/μL), and Nucleic Acid Release Reagent (61202). Specific procedures shall be performed according to the instructions in the kit manual.

**3 Identification of feline parvovirus**

For the identification of feline parvovirus (FPV), FPV identification primers, namely FPV-F: AAAGAGTAGTTGTAAATAA, FPV-R: TATATCACCAAAGTTAGTAG, were synthesized by Nanjing GenScript Biotech Co., Ltd. according to relevant literature reports. The reaction system (20 μL) for FPV identification is shown in the following table1. The PCR reaction consisted of an initial denaturation at 98°C for 1 min, followed by 30 cycles of denaturation at 98°C for 10 s, annealing at 55°C for 5 s, and extension at 72°C for 40 s, and a final extension at 72°C for 10 min. For agarose gel electrophoresis, a 1% agarose gel was prepared by heating to dissolve the agarose, adding 1 μL of Super Red nucleic acid dye, and allowing it to cool. Finally, 2.5 μL of the amplified product was loaded onto the gel for result verification.

Table1 PCR reaction system

| Reagent | Volume（μL） |
| --- | --- |
| Prime STAR HS (Premix) | 10 |
| FPV-F (10μM) | 0.4 |
| FPV-R (10μM) | 0.4 |
| Template | 1 |
| ddH_2_O | 8.2 |

**4 Western Blot**
Total proteins were extracted from FPV virus solution using RIPA lysis buffer (R0010, Solarbio Life Sciences, Beijing, China) supplemented with protease and phosphatase inhibitors. Protein concentration was determined by BCA protein quantification kit, and proteins were separated by 10%-15% SDS-PAGE before being electro-transferred to polyvinylidene fluoride membranes. The membranes were blocked with 5% skim milk in Tris-buffered saline containing Tween 20 (TBST) at room temperature for 1.5 hours, followed by overnight incubation with Anti-Parvovirus (ab140431, Abcam, Cambridge, CA, USA) at 4°C. After washing, the membranes were incubated with horseradish peroxidase-conjugated goat anti-mouse IgG (ab205719, Abcam, Cambridge, CA, USA) at room temperature for 2h. Protein bands were detected using an ECL Western blotting kit (CW0049; Ke Ying Biotechnology Co., Ltd., Beijing), and scanned and analyzed by ImageJ software (National Institutes of Health, Bethesda, MD, USA).

**5 Immunofluorescence Assay**
Fix cells with pre-cooled methanol at -20°C for 10 min, then wash 3 times with pre-cooled PBS. Incubate cells for 30 min with PBST (PBS + 0.1% Tween 20) containing 1% BSA and 22.52 mg/mL glycine. Add Anti-Parvovirus (ab140431, Abcam, Cambridge, CA, USA) diluted in PBST with 1% BSA to cells in a humidified chamber and incubate at 4°C for 16 h. Discard the primary antibody solution, wash cells 3 times with PBST at room temperature, not protected from light, for 5 min each time. Incubate cells with secondary antibody (goat anti-mouse IgG (ab150113, Abcam, Cambridge, CA, USA)) diluted in 1% BSA in the dark at room temperature for 1 h. Discard the secondary antibody solution, wash cells 3 times with PBST at room temperature, protected from light, for 5 min each time. Incubate cells with 10 μg/mL DAPI for 10 min, wash cells 3 times with PBST at room temperature, protected from light, for 5 min each time. Finally, observe under fluorescence microscopy (Zeiss, Germany).

**6 Transmission electron microscopy**
For transmission electron microscopy (TEM) observation: Use a pipette to aspirate 20 μL of the sample and drop it onto a copper grid covered with a carbon film. Let it stand for 3 to 5 minutes, then remove excess liquid with filter paper. Drop 2% phosphotungstic acid onto the copper grid with the carbon support film, let it stand for 1 to 2 minutes, and again remove excess liquid with filter paper. Allow it to dry at room temperature. Observe under a transmission electron microscope (Tecnai G2 F20, FEI Company, USA) and acquire images for analysis.

| 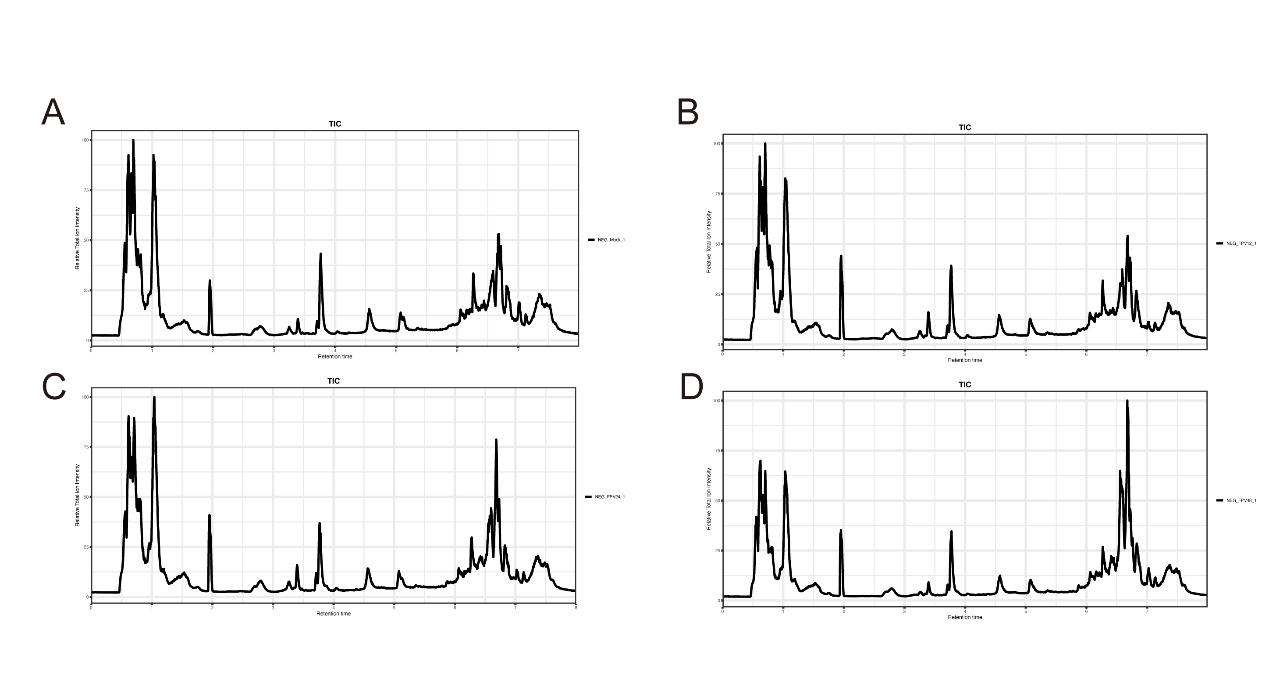 |
| --- |

S1. Liquid-mass profile analysis

1. D. Changes in peak intensity and shape between blank and infected groups at different times

| 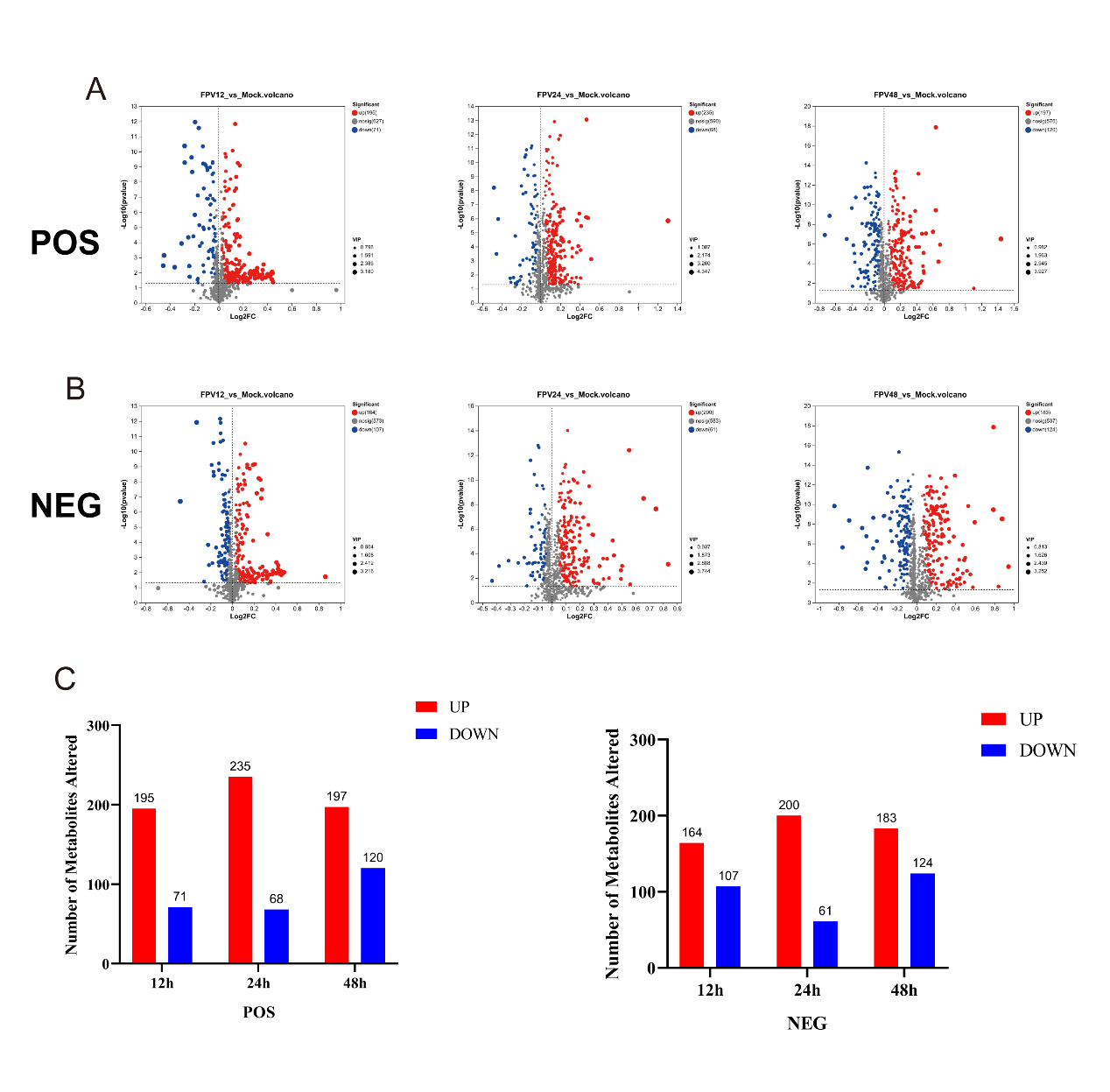 |
| --- |

S2. The number of differential metabolites was counted

1. (POS mode): Scatter plots of FPV12, FPV24, FPV48 vs. mock infected samples in positive ion mode. B. (NEG mode): Scatter plots of FPV12, FPV24, FPV48 vs. mock infected samples in negative ion mode. C. Bar charts showing the number of up - regulated (UP, red) and down - regulated (DOWN, blue) metabolites in POS (left) and NEG (right) modes at 12h, 24h, 48h post infection.

| 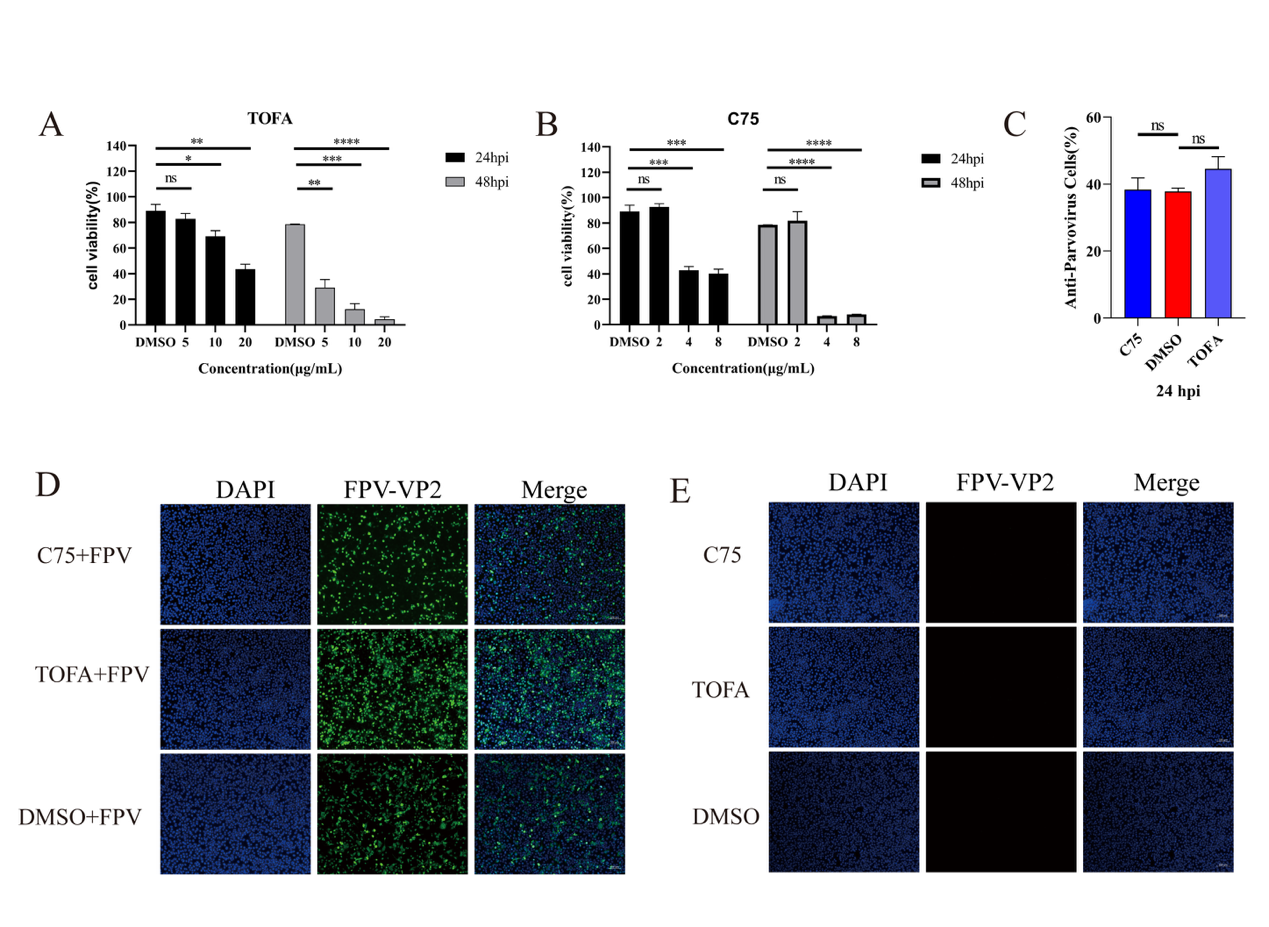 |
| --- |

S3. Effect of fatty acid inhibitors on FPV013 replication

A-B. Detection of cytotoxicity of different concentrations of fatty acid synthase inhibitors (TOFA, C75); C. Indirect immunofluorescence technique was used to detect the expression of FPV VP2, with a scale bar of 100 μm. D. Calculation of the fluorescence ratio. Error bars represent the standard deviation (SD) based on three independent experiments (n=3). Statistical significance was determined using a t-test (*P<0.05; **P<0.01; ***P<0.001; ****P<0.0001).
